# Clinical effect of a dentifrice containing three kinds of bactericidal ingredients on periodontal disease: a pilot study in patients undergoing supportive periodontal therapy

**DOI:** 10.1186/s13104-018-3216-x

**Published:** 2018-02-09

**Authors:** Daichi Kita, Takashi Kinumatsu, Atsushi Yokomizo, Miki Tanaka, Masahiro Egawa, Asako Makino-Oi, Sachiyo Tomita, Atsushi Saito

**Affiliations:** 1 Department of Periodontology, Tokyo Dental College, Tokyo, Japan; 2 Kinumatsu Dental Clinic, Chiba, Japan; 3 Daiichi Sankyo Healthcare Co., Ltd., Tokyo, Japan; 4 Oral Health Science Center, Tokyo Dental College, Tokyo, Japan

**Keywords:** Periodontal disease, Supportive periodontal therapy, Dentifrice, Bactericidal ingredients

## Abstract

**Objective:**

This study aimed to evaluate clinically the effect of a novel dentifrice containing three kinds of bactericidal ingredients on periodontal disease.

**Results:**

This was a single-arm, prospective clinical study that enrolled patients with periodontitis undergoing supportive periodontal therapy. Periodontal examination, microbiological testing of saliva samples, and evaluation of inflammatory markers (IL-1β, IL-6, IL-8, TNF-α) in gingival crevicular fluid were performed. After 4 weeks of the use of test dentifrice, these parameters were re-evaluated. The use of dentifrice was also subjectively evaluated by clinicians and participants. Among 30 participants, there were significant improvements in the periodontal and microbiological parameters, and the level of interleukin-1β in the gingival crevicular fluid, following the use of the test dentifrice. In clinicians’ subjective evaluation of the overall usefulness of the dentifrice, ‘mild’ and ‘moderate’ improvement accounted for 83% of the total responses. In the participants’ subjective evaluation, the majority indicated their experience of the use as favorable. Within the limitations of this study, it is suggested that the progression of periodontal disease during the supportive periodontal therapy can be prevented by the use of the test dentifrice.

*Trial registration* UMIN Clinical Trials Registry (UMIN-CTR) 000023175. Date of formal registration: July 14, 2016 (https://upload.umin.ac.jp/cgi-open-bin/ctr/ctr_view.cgi?recptno=R000026716)

**Electronic supplementary material:**

The online version of this article (10.1186/s13104-018-3216-x) contains supplementary material, which is available to authorized users.

## Introduction

Periodontal diseases are the major cause of loss of teeth [1, 2]. The destruction of periodontal tissue due to the diseases is thought to be caused mainly by inflammation induced by dental plaque consisting of periodontopathogenic bacteria [2]. To prevent periodontal diseases, mechanical plaque control by toothbrush is the most fundamental and effective. However, the mechanical means alone have limitation for the thorough plaque control [3].

In everyday self-care, dentifrices are used as a part of plaque control. It has been reported that the use of dentifrices containing antimicrobial ingredients can enhance the preventive effect on periodontal disease [4, 5]. Medical ingredients such as sodium lauroylsarcosine, isopropyl methylphenol and cetylpyridinium chloride have been shown to be effective in preventing periodontal diseases [4, 5]. However, the effects of combining those ingredients on the prevention of periodontal disease progression have not been clarified.

This study aimed to evaluate the effect of a dentifrice containing the three kinds of bactericidal ingredients on periodontal disease in patients undergoing supportive periodontal therapy (SPT).

## Main text

### Methods

#### Study design

This was a prospective, single-arm clinical study. Participants were recruited from patients with slight or moderate chronic periodontitis [6] who have been receiving SPT at Tokyo Dental College Suidobashi Hospital (Tokyo, Japan) or Tokyo Dental College Chiba Hospital (Chiba, Japan). Sample size was set as 29 patients based on the difference in gingival index (GI) in the previous study using dentifrice [7]; effect quantity: 0.24, standard deviation: 0.5, α-value: 0.05, detection power: 0.80.

#### Inclusion and exclusion criteria

Written informed consent was obtained from all participants. To qualify, patients were required to have a minimum of 20 natural teeth, having at least one site with probing depth (PD) ≥ 4 mm and GI score of 1 or 2. Exclusion criteria were the presence of uncontrolled systemic diseases, smokers, allergy to the dentifrice ingredients, and current pregnancy or lactation. Those with history of active periodontal treatment including scaling and root planning, antimicrobial therapy within 3 months, and contraindications for general dental interventions were excluded. Those who had been already using a previous version of the test dentifrice (Clean Dental^®^, Daiichi Sankyo Healthcare, Tokyo, Japan) are also excluded. The participants received no medication that could affect their periodontal conditions, such as antimicrobial agents or anti-inflammatory drugs, for at least 3 months prior to the study.

#### Periodontal examination

At baseline, trained, calibrated examiners recorded the following: number of sites with gingival swelling, redness, plaque index (PlI), GI, gingival recession, PD and clinical attachment level (CAL) essentially as described previously [8]. These parameters were also recorded at 2 and 4 weeks.

#### Microbiological testing

Saliva samples were collected according to the manufacturer’s instructions and immediately transferred into sampling tubes supplied in a commercial kit (GC, Tokyo, Japan) and sent to a commercial testing laboratory (GC Oral Check Center, Tokyo, Japan: http://www.gcoc.jp/). The numbers (copies) of periodontal pathogens (*Porphyromonas gingivalis*, *Aggregatibacter actinomycetemcomitans*, *Treponema denticola*, *Tannerella forsythia*, *Prevotella intermedia*) and the total bacteria in saliva were measured by using real-time polymerase chain reaction method.

#### Assessment of inflammatory markers

Sampling of gingival crevicular fluid (GCF) was performed by inserting a paper strip into the gingival sulcus of one tooth with a deep pocket. Samples were sent to a commercial testing laboratory (GeneticLab, Sapporo, Japan). Measurement of inflammatory markers (IL-1β, IL-6, IL-8, TNF-α) in GCF was performed by the laboratory. Briefly, the protein was extracted from the paper strip and the total protein content (mg/mL) in the extraction solution was measured by a colorimetric method. After labeling of each inflammatory marker, the amount in the extraction solution (pg/mL) was calculated by measuring the fluorescence intensity. Finally, the amount of each inflammatory marker (pg/mg) to the total protein mass of 1 mg was calculated by dividing the amount of the inflammatory marker in GCF (pg/mL) by the total protein content (mg/mL).

#### Use of test dentifrice

A novel dentifrice (OC 1510, provisional name: Clean Dental^®^ product under development, Daiichi Sankyo Healthcare) containing 10 medicinal ingredients including 3 bactericidal agents (sodium lauroylsarcosine, isopropyl methylphenol and cetylpyridinium chloride) and 2 anti-inflammatory agents (β-glycyrrhetinic acid and ε-aminocaproic acid) was used for the study. Participants were asked to use 1 g of the test dentifrice and brush with the scrubbing method 3 times/day after meals for 4 weeks. A standard toothbrush (Dent EX Systema 44M, Lion, Japan) was supplied to each participant. A leaflet describing the toothbrushing method using the test dentifrice was distributed to each participant. In order to monitor compliance, they were asked to record the use of dentifrice.

#### Subjective evaluation of overall usefulness by clinicians

At the end of the study, supervising clinicians evaluated the participants’ overall usefulness of the test dentifrice relative to baseline conditions, using a Likert scale: 5; marked improvement, 4; moderate improvement, 3; mild improvement, 2; no difference, 1: worse.

#### Subjective evaluation by participants

A questionnaire survey on the use of test dentifrice was carried out. The questionnaire items are shown in Additional file 1: Table S1.

#### Statistical analysis

For statistical analysis, GraphPad InStat version 3.10 (GraphPad Software, Inc. La Jolla, CA) was used. Friedman test with Dunn post-hoc test was used, and p-values less than 0.05 were considered statistically significant.

### Results

#### Demographic information on participants

In this study, a total of 30 chronic periodontitis patients (9 males and 21 females, mean 58.6 years old, range: 32–70 years old) were enrolled and completed the study.

#### Periodontal examination

The results of periodontal examination are shown in Table 1. Significant improvements were observed in gingival redness, PlI and GI at 2 weeks and 4 weeks compared to baseline (p < 0.05). At 2 weeks, no significant improvement from baseline was observed in gingival swelling, PD, CAL, and GI ≥ 2 sites. However, significant improvement was observed at 4 weeks (p < 0.01). In the amount of gingival recession, significant difference from baseline and 2 weeks was noted at 4 weeks (p < 0.001).

**Table 1:** Changes in periodontal parameters

	Baseline	2 weeks	4 weeks
Gingival swelling	0.22 ± 0.35	0.11 ± 0.22	0.06 ± 0.12**
Gingival redness	0.32 ± 0.41	0.19 ± 0.33*	0.16 ± 0.24***
PlI	0.31 ± 0.32	0.18 ± 0.18**	0.17 ± 0.18**
GI	0.31 ± 0.27	0.22 ± 0.25*	0.17 ± 0.17***
Gingival recession (mm)	0.56 ± 0.50	0.56 ± 0.52	0.61 ± 0.54***,^†††^
PD (mm)	2.24 ± 0.27	2.17 ± 0.26	2.10 ± 0.29***
CAL (mm)	2.80 ± 0.51	2.73 ± 0.58	2.71 ± 0.54**
Number of sites with GI ≥ 2	16.33 ± 15.86	11.70 ± 12.65	9.10 ± 10.27***

Data shown as mean ± standard deviation. * p < 0.05, ** p < 0.01, *** p < 0.001 vs. baseline, ^†††^ p < 0.001 vs. 2 weeks

Throughout the study, no serious adverse events were reported.

#### Microbiological findings

The results of the microbiological testing are shown in Table 2. The copy numbers of *T. denticola* and *T. forsythia* were significantly decreased at 4 weeks compared to baseline (p < 0.05). No significant change was observed in other bacteria for 4 weeks. Total copy numbers of bacteria were significantly decreased at 2 weeks compared to baseline (p < 0.01), but no significant difference was observed at 4 weeks.

**Table 2:** Number of bacteria; log copies (ratio of each bacteria to the total number; ×10^−3^ %)

	Baseline	2 weeks	4 weeks
Total bacteria	9.24 ± 9.08	9.01 ± 8.92**	9.04 ± 8.87
*Porphyromonas gingivalis*	5.89 ± 6.35 (37.48 ± 95.97)	5.26 ± 5.55 (17.21 ± 30.54)	5.46 ± 5.93 (20.09 ± 39.56)
*Aggregatibacter actinomycetemcomitans*	3.06 ± 3.63 (0.12 ± 0.48)	2.87 ± 3.37 (0.14 ± 0.61)	2.80 ± 3.30 (0.10 ± 0.39)
*Treponema denticola*	4.52 ± 4.65 (2.15 ± 2.62)	4.46 ± 4.66 (2.51 ± 3.61)	4.27 ± 4.47* (1.49 ± 2.09)
*Tannerella forsythia*	4.74 ± 4.92 (3.15 ± 4.04)	4.66 ± 4.88 (4.02 ± 6.77)	4.69 ± 5.01* (3.55 ± 5.82)
*Prevotella intermedia*	5.49 ± 5.98 (18.29 ± 54.65)	5.94 ± 6.60 (60.61 ± 291.80)	5.99 ± 6.68 (62.60 ± 310.50)

Data shown as mean ± standard deviation. * p < 0.05, ** p < 0.01 vs. baseline

#### Inflammatory markers in GCF

The measurement results of inflammatory markers in GCF are shown in Additional file 2: Table S2. The sample size of IL-6 was smaller than other cytokines, because the median values of the obtained fluorescence intensity were too low for calculating the concentration in some samples.

Among the four cytokines, a significant reduction from baseline was observed for IL-1β at 2 weeks (p < 0.05). No significant difference was observed between 2 weeks and 4 weeks. In other cytokines, no significant change was observed during the test period.

#### Overall usefulness

“Mild improvement” was reported by 60% (18/30) of clinicians, followed by “moderate improvement” 23.3% (7/30), and “no difference” 16.7% (5/30).

#### Subjective evaluation by the participants

**Fig. 1:**
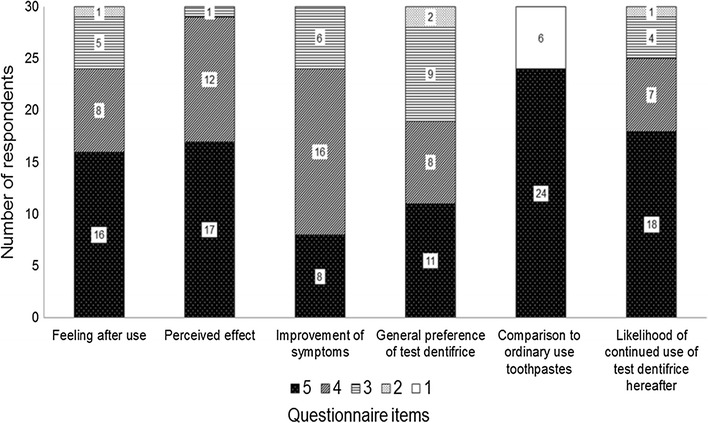
Subjective evaluation by participants: distribution of responses

The questionnaire results for the use of test dentifrice are shown in Fig. 1. In response to “feeling after use”, 24 (80%) participants scored 4 (good) or 5 (very good). In response to “perceived effect”, 29 (96.7%) participants scored 4 or 5. As for “improvement of symptoms”, 24 (80%) participants scored 4 (slight improvement) or 5 (much improvement). In response to “general preference of test dentifrice”, 19 (63.3%) participants scored 4 (somewhat preferred) or 5 (much preferred), while two (6.7%) scored 2 (somewhat dispreferred). As for “comparison to ordinary use toothpastes”, 24 (80%) of participants scored 5 (much preferred), but 6 (20%) participants scored 1 (dispreferred). In response to “likelihood of continued use of test dentifrice hereafter”, 25 (83.3%) participants scored 4 (probably) or 5 (definitely), but one (3.3%) scored 2 (probably not). Overall, those who scored 4 or 5 accounted for 63% or more in questionnaire items.

### Discussion

In the present study, we investigated the effect of the test dentifrice, which contains sodium lauroylsarcosine, isopropyl methylphenol and cetylpyridinium chloride and other medicinal ingredients, on periodontal conditions of patients undergoing SPT. Following the use of the test dentifrice for 4 weeks, significant improvements from baseline were observed in all periodontal parameters. No significant adverse events were noted. These findings suggest that this dentifrice can be safely used to prevent the progression of periodontitis during SPT. It has been reported that gingivitis is alleviated by using dentifrices containing β-glycyrrhetinic acid [10, 11], ε-aminocaproic acid [9, 10] or sodium chloride [9, 10]. Improvement in periodontal conditions observed in this study can be attributed to any of these and the bactericidal ingredients, because the test dentifrice contains all of them. In the subjective evaluation of overall usefulness, 60% of clinicians reported “mild improvement”. This may be attributed to the fact that the periodontal conditions at baseline were relatively stable for these participants because they were already in the SPT phase.

As for the microbiological testing, we targeted the representative periodontal pathogens in saliva. Among them, *P*. *gingivalis*, *T*. *denticola* and *T*. *forsythia* belong to the ‘red complex’ bacterial species [12], and their close associations with periodontitis have been reported [13, 14]. In this study, the numbers of *T*. *denticola* and *T*. *forsythia* significantly decreased at 4 weeks compared to baseline. This finding suggests that the test dentifrice may exert a suppressing effect on the growth of the periodontal pathogens. Sodium lauroylsarcosine is an anionic surfactant [4]. Anionic surfactants have been shown to exert a bactericidal effect due to its ability of destroying bacterial cell membranes and denaturing bacterial proteins. Isopropyl methylphenol has been reported to penetrate biofilm and exert bactericidal action [15]. In an ex-vivo experiment, a mouthrinse containing 0.05% cetylpyridinium chloride demonstrated a significant antimicrobial activity on plaque microorganisms [16]. These ingredients in the test dentifrice might have contributed to suppressing the periodontal pathogens.

In the assessment of inflammatory markers in GCF, IL-1β was found to be significantly decreased at 2 weeks compared to baseline. This suggests that this dentifrice has a potential effect of suppressing gingival inflammation from the relatively early stage of use. An in vitro study showed that the production of IL-1β was decreased by dentifrices containing triclosan [17, 18]. However, the test dentifrice in the present study does not include triclosan. Information is limited on the relationship between the dentifrice ingredients and the dynamics of inflammatory markers in periodontal milieu. Further investigation is necessary for understanding the true effect of test dentifrice ingredients on the release of inflammatory markers.

With regard to the subjective evaluation of the use of test dentifrice by participants, their responses for items such as feeling of use (taste, smell), feeling of effect (sense of refreshing and its duration), or whether to use in the future, were generally favorable. On the other hand, regarding the improvement of the symptoms and preference of this product, the responses were somewhat different. It may have been difficult for the participants to be conscious of a potential change in their oral conditions, compared to patients who are undergoing active periodontal treatment. Furthermore, the reason for relatively poor score on the preference of the test dentifrice may be attributed to somewhat salty flavor of the test dentifrice.

### Conclusions

Significant improvements were found in the periodontal parameters, microbiological findings in saliva and IL-1β in GCF, following the 4-week use of the novel dentifrice containing 3 kinds of bactericidal ingredients. Both clinicians’ and participants’ perceptions of the use of the test dentifrice were generally favorable. The data collectively suggest that the progression of periodontal disease during SPT may be prevented by the use of this dentifrice.

## Limitations

This was a single-arm, prospective, non-randomized study with no control group. Since the sample size is small, it is difficult to draw definite conclusions or broad generalizations from this study. It is also difficult to interpret how subjective the clinician and patient reporting is.

A randomized controlled trial using a larger sample size is necessary to clarify the true clinical benefit of the test dentifrice.

## Additional files

**Additional file 1: Table S1.** Subjective evaluation by participants: Questionnaire items.

**Additional file 2: Table S2-1.** The amounts of inflammatory markers in GCF (pg/mL). **Table S2-2.** The amounts of each inflammatory markers to 1 mg total protein (pg/mg).

## Abbreviations

SPT: supportive periodontal therapy
PD: probing depth
GI: gingival index
PlI: plaque index
CAL: clinical attachment level
GCF: gingival crevicular fluid
