# Supplementary material for: Clinical effect of a dentifrice containing three kinds of bactericidal ingredients on periodontal disease: a pilot study in patients undergoing supportive periodontal therapy
Source: BMC Res Notes. 2018 Feb 9;11:116. doi: 10.1186/s13104-018-3216-x (PMC5807746; doi:10.1186/s13104-018-3216-x)
Supplement: Supplementary file 1 — Additional file 1: Table S1. Subjective evaluation by participants: Questionnaire items. [file 13104_2018_3216_MOESM1_ESM.pdf]

Table S1 Subjective evaluation by participants: Questionnaire items

Feeling after use	5: Very good 4: Good 3: Acceptable 2: Poor 1: Very poor
Perceived effect	5: Very good 4: Good 3: Acceptable 2: Poor 1: Very poor
Improvement of symptoms	5: Much improvement 4: Slight improvement 3: Neither 2: Not so much improvement 1: No improvement
General preference of test dentifrice	5: Much preferred 4: Somewhat preferred 3: Neither 2: Somewhat dispreferred 1: Dispreferred
Comparison to ordinary use toothpastes	5: Much preferred 4: Somewhat preferred 3: Neither 2: Somewhat dispreferred 1: Dispreferred
Likelihood of continued use of the test dentifrice hereafter	5: Definitely 4: Probably 3: Neither 2: Probably not 1: Definitely not
