# Supplementary material for: Clinical effect of a dentifrice containing three kinds of bactericidal ingredients on periodontal disease: a pilot study in patients undergoing supportive periodontal therapy
Source: BMC Res Notes. 2018 Feb 9;11:116. doi: 10.1186/s13104-018-3216-x (PMC5807746; doi:10.1186/s13104-018-3216-x)
Supplement: Supplementary file 2 — Additional file 2: Table S2-1. The amounts of inflammatory markers in GCF (pg/mL). Table S2-2. The amounts of each inflammatory markers to 1 mg total protein (pg/mg). [file 13104_2018_3216_MOESM2_ESM.pdf]

Table S2-1 The amount of inflammatory markers in GCF (pg/mL)

	Baseline	2 weeks	4 weeks
IL-1β	46.46 ± 105.74	29.19 ± 69.41*	16.59 ± 35.79
IL-6	2.14 ± 3.69	0.51 ± 0.40	0.92 ± 1.09
IL-8	670.38 ± 945.51	399.08 ± 526.31	314.32 ± 467.46
TNF-α	2.44 ± 2.60	1.37 ± 1.28	1.57 ± 1.93

Data shown as mean ± standard deviation (n=30, except for IL-6; n=17). *: $p<0.05$ vs. baseline.

Table S2-2 The amount of each inflammatory marker to 1 mg total protein (pg/mg)

	Baseline	2 weeks	4 weeks
IL-1β	102.79 ± 138.70	120.39 ± 263.56	106.48 ± 161.16
IL-6	22.84 ± 63.36	6.52 ± 7.45	7.04 ± 6.01
IL-8	2979.74 ± 2835.12	2901.98 ± 3112.92	2426.30 ± 3418.78
TNF-α	21.04 ± 33.15	16.64 ± 16.18	16.99 ± 18.54

Data shown as mean ± standard deviation (n=30, except for IL-6; n=17).
